# Treatment of advanced neuroblastoma: feasibility and therapeutic potential of a novel approach combining 131-I-MIBG and multiple drug chemotherapy

**DOI:** 10.1054/bjoc.2000.1645

**Published:** 2001-02

**Authors:** S Mastrangelo, A Tornesello, L Diociaiuti, A Pession, A Prete, V Rufini, L Troncone, R Mastrangelo

**Affiliations:** Pediatric Oncology; Dept. Nuclear Medicine, Catholic University, Rome, Italy; Dept. Paediatrics III, Bologna, Italy

**Keywords:** neuroblastoma, combined therapy, 131-I-metaiodobenzylguanidine

## Abstract

Biological and clinical observations suggest that initial marked reduction of resistant clones may be critical in any attempt to improve long-term results in advanced neuroblastoma (NB). The aim of this pilot study is to determine short-term toxicity and efficacy of a new therapeutic model based on the simultaneous use of multiple drug chemotherapy and specific irradiation using 131-I-MIBG. The study population consisted of 21 patients, from 1 to 8 years of age with good 131-I-MIBG uptake. 16 extensively pre-treated patients with refractory or relapsed disease were divided into 2 groups. In Group 1 (9 patients) the basic chemotherapy regimen consisted in cisplatin at the dose of 20 mg/m^2^i.v. per day infused over 2 h, for 4 consecutive days; on day 4 Cy 2 g/m^2^i.v. was administered over 2 h followed by Mesna. Group 2 (7 patients) was treated with basic chemotherapeutic regimen plus VP16 and Vincristine. VP16 at the dose of 50 mg/m^2^i.v. per day was administered as a 24 h infusion on days 1–3; Vincristine 1.5 mg/m^2^i.v. was administered on days 1 and 6. On day 10 a single dose of 131-I-MIBG (200 mCi) with a high specific activity (>1.1 GBq/mg) was administered to both Groups by i.v. infusion over 4–6 hours. A further 5 patients were treated at diagnosis: 2 with the same regimen as Group 1 and 3 with the same as Group 2. The severity of toxicity was graded according to World Health Organization (WHO) criteria. Assessment of tumour response was monitored 4–6 weeks after the beginning of combined therapy (CO-TH). Response was defined according to INSS (International Neuroblastoma Staging System) criteria. No extra-medullary toxicity was observed in any patient. Haematological toxicity was the only toxicity observed and seemed mainly related to chemotherapy. Myelosuppression was mild in the 5 patients treated at diagnosis. No serious infections or significant bleeding problems were observed. In the 16 resistant patients, 12 PR, 1 mixed response and 3 SD were obtained. In the 5 patients treated at diagnosis 2 PR, 1 CR and 2 VGPR were observed. No alteration in 131-I-MIBG uptake was observed after the chemotherapy preceding radio-metabolic treatment. The therapeutic results of this pilot regimen of CO-TH resulted in a high percentage of major response after only a single course in both resistant patients and patients treated at diagnosis. Because of the minimal toxicity observed in patients studied at diagnosis so far, there is room for gradual intensification of the treatment. It is to be hoped that this suggested novel approach may represent an important route of investigation to improve final outcome in patients with advanced NB. © 2001 Cancer Research Campaign http://www.bjcancer.com


*British Journal of Cancer* (2001) **84**(4), 460–464
© 2001 Cancer Research Campaign
doi: 10.1054/ bjoc.2000.1645, available online at http://www.idealibrary.com on IDEAL®

http://www.bjcancer.com

### Treatment of advanced neuroblastoma: feasibility and therapeutic potential of a novel approach combining 131-I-MIBG and multiple drug chemotherapy

**S Mastrangelo[1], A Tornesello[1], L Diociaiuti[1], A Pession[3], A Prete[3], V Rufini[2], L Troncone[3] and R Mastrangelo[1]**

[1]Pediatric Oncology, [2]Dept. Nuclear Medicine, Catholic University, Rome; [3]Dept. Paediatrics III, Bologna, Italy

**Summary** Biological and clinical observations suggest that initial marked reduction of resistant clones may be critical in any attempt to improve long-term results in advanced neuroblastoma (NB). The aim of this pilot study is to determine short-term toxicity and efficacy of a new therapeutic model based on the simultaneous use of multiple drug chemotherapy and specific irradiation using 131-I-MIBG. The study population consisted of 21 patients, from 1 to 8 years of age with good 131-I-MIBG uptake. 16 extensively pre-treated patients with refractory or relapsed disease were divided into 2 groups. In Group 1 (9 patients) the basic chemotherapy regimen consisted in cisplatin at the dose of 20 mg/m$^2$ i.v. per day infused over 2 h, for 4 consecutive days; on day 4 Cy 2 g/m$^2$ i.v. was administered over 2 h followed by Mesna. Group 2 (7 patients) was treated with basic chemotherapeutic regimen plus VP16 and Vincristine. VP16 at the dose of 50 mg/m$^2$ i.v. per day was administered as a 24 h infusion on days 1–3; Vincristine 1.5 mg/m$^2$ i.v. was administered on days 1 and 6. On day 10 a single dose of 131-I-MIBG (200 mCi) with a high specific activity (>1.1 GBq/mg) was administered to both Groups by i.v. infusion over 4–6 hours. A further 5 patients were treated at diagnosis: 2 with the same regimen as Group 1 and 3 with the same as Group 2. The severity of toxicity was graded according to World Health Organization (WHO) criteria. Assessment of tumour response was monitored 4–6 weeks after the beginning of combined therapy (CO-TH). Response was defined according to INSS (International Neuroblastoma Staging System) criteria. No extra-medullary toxicity was observed in any patient. Haematological toxicity was the only toxicity observed and seemed mainly related to chemotherapy. Myelosuppression was mild in the 5 patients treated at diagnosis. No serious infections or significant bleeding problems were observed. In the 16 resistant patients, 12 PR, 1 mixed response and 3 SD were obtained. In the 5 patients treated at diagnosis 2 PR, 1 CR and 2 VGPR were observed. No alteration in 131-I-MIBG uptake was observed after the chemotherapy preceding radio-metabolic treatment. The therapeutic results of this pilot regimen of CO-TH resulted in a high percentage of major response after only a single course in both resistant patients and patients treated at diagnosis. Because of the minimal toxicity observed in patients studied at diagnosis so far, there is room for gradual intensification of the treatment. It is to be hoped that this suggested novel approach may represent an important route of investigation to improve final outcome in patients with advanced NB. © 2001 Cancer Research Campaign http://www.bjcancer.com

**Keywords**: neuroblastoma; combined therapy; 131-I-metaiodobenzylguanidine

For many years there has been no substantial improvement in survival of children with advanced neuroblastoma (NB) >1 year of age (Matthay et al, 1996), despite the increasingly aggressive therapies employed. To date, the most common treatment for such patients comprises induction chemotherapy over a period of several months, usually followed by high-dose consolidation chemotherapy with the aim of destroying eventual residual tumour cells resistant to induction chemotherapy. A better therapeutic approach could be the development of intensive, multiple drug, multiple modality therapy protocols at diagnosis in order to rapidly destroy drug-resistant clones present ab initio and prevent emergence of drug-resistant mutants.

131-I-metaiodobenzylguanidine (131-I-MIBG), a radio-iodinated aralkylguanidine, is a derivative of the neuron-blocking agent guanethidine, which is capable of competing with norepinephrine for an active uptake into neuroadrenergic tissue and derived tumours (Wieland et al, 1980; Ivarone et al, 1993). Originally employed for phaeochromocytoma imaging, 131-I-MIBG was subsequently used for NB (Geatti et al, 1985). Owing to its concentration in NB lesions, 131-I-MIBG has the potential for specifically delivering very large radiation doses to NB malignant cells, and NB is known to be a radio-sensitive tumour.

Up to now, 131-I-MIBG alone has mostly been used in patients resistant to conventional therapy with a partial remission achieved in more than 30% of cases (Mastrangelo, 1987; Matthay et al, 1998). The only significant toxicity was myelosuppression. Thus, 131-I-MIBG shows a response rate in NB similar to, if not better than, the most active chemotherapeutic agents. Good results, with mild haematological toxicity, have also been obtained using 131-I-MIBG alone at diagnosis. (Mastrangelo et al, 1989; Mastrangelo and Voute, 1991; Mastrangelo et al, 1993; Dekraker et al, 1995).

The final objective of the work we have underway is to develop a regimen for advanced NB to be adopted at diagnosis, based on the simultaneous use of all available drugs, known to be effective against NB, in combination with 131-I-MIBG. In the present study response rates, and principally, ways of overcoming potential toxicity of CO-TH have been investigated. We have studied this novel therapeutic approach first in resistant NB patients and then in a number of cases with advanced disease at diagnosis.

*Received 16 June 2000*
*Revised 25 September 2000*
*Accepted 21 November 2000*

*Correspondence to:* R Mastrangelo

### PATIENTS AND METHODS

From 1996 to date, 21 patients ≥1 year of age with advanced NB, underwent CO-TH. 16 with refractory or relapsed disease and 5 patients with advanced disease at diagnosis were admitted to the study. Informed parental consent was obtained in all cases.

16 patients (11 males, 5 females), aged 1 to 8 years (median 5 years), with relapsed or refractory NB were treated. All patients showed evaluable disease and a good 131-I-MIBG uptake at enrolment. The median interval between the last day of prior treatment and the beginning of CO-TH was 3 months (range 1–12 months); most patients were treated following recovery from previous toxicity. All were heavily pre-treated with multiple courses of combined agents including platinum compounds, cyclophosphamide (Cy), VP-16 and Vincristine (VCR). Group 1 included 9 patients treated with a regimen based on cisplatin and Cy administered before 131-I-MIBG. Subsequently, in the second group of 7 patients (Group 2) VP16 and VCR were included in the chemotherapy regimen. Patients in both groups were comparable in regard to intensity of prior treatment.

5 patients with newly diagnosed advanced NB with a good 131-I-MIBG uptake were treated with CO-TH. There were 4 males and 1 female, ranging in age from 2 to 4 years; 4 presented with Stage 4 disease and one with a non-resectable Stage 3 NB. In patients 2 and 5, bone marrow was heavily infiltrated by neuroblastoma cells.

### Evaluation

Method for assessment of disease extent just before and following CO-TH included: computerised tomography (CT) or nuclear magnetic resonance (NMR) imaging of tumour mass and 131-I-MIBG or 123-I-MIBG scan; technetium-99 m scan and/or X-rays were also used for evaluation of bone disease. Bone marrow aspiration from four different sites on the iliac crest and biopsy specimens from two sites completed the evaluation. 24 urine collections were carried out for catecholamines and catecholamine metabolites. Cardiac and kidney functions were also investigated and audiograms were performed, prior to and following CO-TH; evaluation of toxicity was scheduled, including ECG and echocardiography for cardiac function; renal function was assessed by 24 h creatinine clearance and sequential renal angioscintigraphy. Haematological toxicity was assessed by performing complete blood cell counts 2–3 times a week. Severity of toxicity was graded according to World Health Organization criteria (WHO, 1979). Assessment of tumour response was monitored 4 to 6 weeks after the beginning of CO-TH. Response was defined according to INSS criteria (Brodeur et al, 1993). Semi-quantitative measurement of 131-I-MIBG uptake was carried out on planar images by calculating tumour-to-background ratios using the region of interest (ROI) analysis. Radionuclide uptake was expressed in the ratio of average counts of pixels in the tumour ROI to average counts of pixels in background ROI (drawn in a region adjacent to the tumour).

### Treatment regimen

An outline of the final CO-TH regimen is shown in Figure 1. The basic chemotherapy regimen (Group 1) consisted in cisplatin at the dose of 20 mg/m$^2$ per day given as a 2 h i.v. infusion for 4 consecutive days; on day 4 cyclophosphamide (Cy) 2 g/m$^2$ i.v. was administered over 2 h followed by Mesna. In patients from Group 2, VP16 and VCR were also included (regimen 2). VP16 at the dose of 50 mg/m$^2$ i.v. per day was given as a 24 h infusion on days 1–3; vincristine, 1.5 mg/m$^2$ i.v., was administered on days 1 and 6. On day 10 a single dose of 131-I-MIBG (200 mCi) with a high specific activity (>1.1 GBq/mg) was given by i.v. infusion over 4–6 hours. A reduced dose of 100 mCi was planned for children under 2 years of age. Radiation dosimetry was not performed. Iodine solution for thyroid blocking was commenced 3 days before 131-I-MIBG infusion and continued for 3 weeks. Post-therapeutic 131-I-MIBG imaging was recorded weekly.

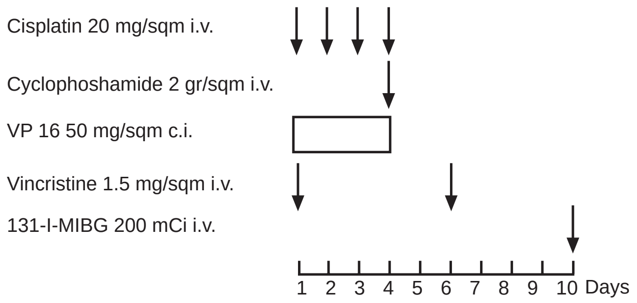

**Figure 1** Combined treatment regimen.

The first 2 patients treated at diagnosis received CO-TH regimen 1; the last 3 patients received regimen 2

### RESULTS

### Toxicity

The 21 patients submitted to CO-TH did not show any acute side-effects directly related to 131-I-MIBG administration apart from occasional vomiting and a single episode of mild acute haemorrhagic cystitis. No extra-medullary toxicity was observed from the beginning of treatment and during the study period. Specifically, no changes were detectable in renal or cardiac function; on the whole, the general condition of these children has been excellent.

In the resistant patients of Groups 1 and 2, a comparable haematological toxicity was observed. In most patients, a nadir in the absolute neutrophil count (ANC) of below 500/mm$^3$ occurred between day 6 and day 20 with a duration of 3–15 days; a nadir in the platelet (PLT) count of below 50 000 occurred on approximately day 15 and lasted 2–5 days. In patients who had received melphalan at standard dosage within 2 months prior to CO-TH, a prolonged neutropenia and thrombocytopenia were observed. However, no serious infections or bleeding tendencies were observed in any of the patients investigated. Figure 2 shows in detail the time course of neutrophil and platelet mean values in patients of Groups 1 and 2 combined.

In the 5 patients treated at diagnosis, myelosuppression was relatively mild and appeared to be related solely to the effect of the chemotherapeutic agents. Despite ANC even of below 500/mm$^3$ at the time of 131-I-MIBG administration, recovery from myelosuppression was rapid in cases 1, 3 and 4 (range 7–10 days). In cases 2 and 5, where there was massive BM infiltration, haematological recovery was somewhat less rapid, since ANC and platelet counts were respectively >2000 and >150 000 mm$^3$ 3 weeks after 131-I-MIBG administration. Although these are preliminary data, haematological toxicity from subsequent courses of chemotherapy appears comparable to that experienced by patients with advanced

*© 2001 Cancer Research Campaign*

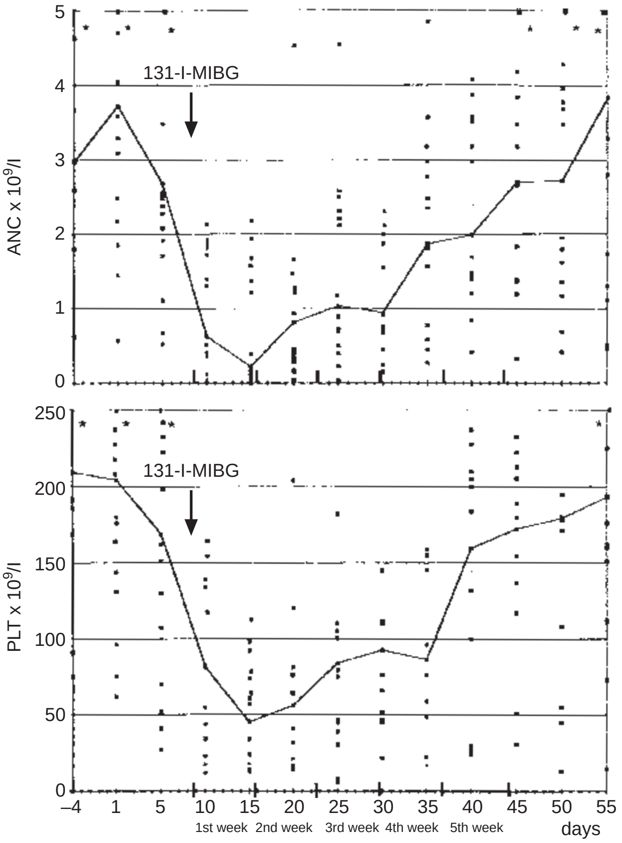

**Figure 2** Time course of absolute neutrophil (ANC $\times$ $10^9$/l) (a) and platelet counts (PLT $\times$ $10^9$/l) (b) following CO-TH (*data over line). Individual and mean values are shown. The arrow indicates days of 131-I-MIBG administration.

NB treated in our centre with identical drug combinations but without prior CO-TH.

### Clinical response

The overall response rate in Groups 1 and 2 combined shows 12 PR (75%), I mixed response and 3 SDs (see Tables 1 and 2). The median duration of progression-free survival from the beginning of CO-TH was 12 months (range 3–42 months). 3 patients are in continuous complete remission: patient 5 (Table 1) and patient 15 (Table 2) are in continuous CR respectively at 35 and 28 months following surgery, patient 7 (Table 1) following surgery and BMT at 42 months. The high response rate was observed even in patients who appeared to be previously resistant to drugs used in CO-TH.

Clinical response, evaluated 4–5 weeks following the beginning of CO-TH, of patients treated at diagnosis, included 2 PR 1 CR and 2 VGPR (see Table 3). In particular, the CT scan of case 3 showed, 5 weeks from start of treatment, an almost 100% reduction of the primary tumour, while the 131-I-MIBG scan was completely negative. BM and catecholamines were normal.

Results of semi-quantitative measurement of 131-I-MIBG uptake are shown in Table 4. No evidence of modified uptake was observed in the 6 patients (four at diagnosis) investigated before and after treatment with 2- and 4-drug chemotherapy, just before 131-I-MIBG administration.

### DISCUSSION

Current treatment of advanced neuroblastoma (NB) has so far produced a poor prognosis. Results of even intensive chemotherapy are disappointing as compared to other childhood malignant tumours. In advanced NB, as with ALL and B lymphoma, it may be essential to increase the proportion of patients achieving a CR in a

**Table 1** Patients' characteristics and results of treatment in Group 1 resistant NB

| Pt. | Sex/Age | Disease extent | | | Previous treatment | Disease status | Response |
|---|---|---|---|---|---|---|---|
| | | Soft tissues | BM | Boney lesions | | | |
| 1 | M/2 | Abdominal mass mediastinic mass | 30% | Humerus | De CECAT (4) HDCY-Adr (4) Cis-131-I-MIBG (2) | 2nd relapse PD | PR |
| 2 | F/6 | Cerebral localization | – | – | De CECAT (6) ABMT (Bu-CP16-Thio) | 1st relapse | PR |
| 3 | M/3 | Abdominal mass Hepatic lesions | – | – | PTC (2) De CECAT (4) HDCY-Adr (2) ABMT (Bu-CY-Melph) | 1st relapse PD | PR |
| 4 | M/3 | Abdominal mass | 10% | Pelvis Femur Rib | 131-I-MIBG (2) CaDO (2) Carbo-VP16 (2) | 1st relapse PD | PR |
| 5 | F/3 | Adrenal mass | – | – | De CECAT (4) | Refractory | SD |
| 6 | M/3 | – | 30% | Femur Cnemis | De CECAT (3) HDCY-Adr (2) Melph (2) | Refractory | Mixed Response |
| 7 | M/5 | Adrenal mass | 4% | Pelvis | De CECAT (4) HDCY-Adr (2) Melph (1) | Refractory | PR |
| 8 | M/8 | Mediastinic mass | – | – | De CECAT (4) ABMT (Bu-VP16-Thio) Melph (3) | Refractory | PR |
| 9 | F/5 | Adrenal mass | – | 1st throracic vertebra Rib | De CECAT (4) HDCY-Adr (2) | Refractory | SD |

 *© 2001 Cancer Research Campaign*

**Table 2** Patients' characteristics and results of treatment in Group 2 resistant NB

| Pt. | Sex/Age | Disease extent | | | Previous treatment | Disease status | Response |
|---|---|---|---|---|---|---|---|
| | | **Soft tissue** | **BM** | **Bony lesion** | | | |
| 10 | F/7 | Cerebral Localization | 90% | Lumbar rachis Rib Pelvis | De CECAT (4) ABMT (Bu-VP16-Thio) 131-I-MIBG (2) | 2nd relapse PD | PR |
| 11 | M/4 | Adrenal mass Mediastinic mass | – | Lumbar rachis | De CECAT (6) HDCY-Adr (2) | Refractory | PR |
| 12 | M/3 | Adrenal Mediastinal mass | 3% | – | De CECAT (4) HDCY-VP16 (2) | Refractory | SD |
| 13 | M/1 | Mediastinic mass | – | – | CY-VP16 (2) CY-Adr (1) Cis-131-I-MIBG (1) | Refractory | PR |
| 14 | M/6 | Adrenal mass Hepatic mass Thoracic lesions | – | – | CY-Adr (2) Carbo–VP16 (2) CY-CP16 (1) ABMT (CARBO-VP16-Thio-Melph) | Refractory | PR |
| 15 | M/2 | Adrenal mass | – | – | IFO-Adr (2) CY-VP16 (2) HDCY 2) | Refractory | PR |
| 16 | F/1 | Abdominal mass | – | – | CARBO-VP16 (2) IFO-Adr (2) CY-VP16 (2) ABMT (Thio-Melph) (CY-Thio) (Melph) | 1st relapse | PR |

**Table 3** Patients' characteristics and results of treatment in advanced NB at diagnosis

| Pt. | Sex/Age | Disease extent Soft tissue | BM | Cortical bony lesions | Regimen Combined therapy (single course) | Response (after 4–5 weeks) |
|---|---|---|---|---|---|---|
| 1 | M/3 | +++ | + | +++ | Cis-Cy---131-I-MIBG | PR |
| 2 | F/3 | +++ | +++ | +++ | Cis-Cy---131-I-MIBG | PR |
| 3 | M/2 | +++ | ++ | +++ | Cis-Cy-VP16-VCR-131-I-MIBG | CR |
| 4^ | M/4 | +++ | – | – | Cis-Cy-VP16-VCR-131-I-MIBG | VGPR |
| 5 | M/3 | ++ | +++ | ++ | Cis-Cy-VP16-VCR-131-I-MIBG | VGPR |

^3rd stage short time. Long-term survivors are rare in advanced NB, and they are almost invariably patients in whom a CR has been rapidly obtained (Simone, 1984). A higher percentage of long-term survivors may also depend on a better quality of first remission, since recurrence, for the present, is frequent.

The present study describes a new combination therapy (CO-TH) in advanced NB, which uses, in resistant patients as well as in patients at diagnosis, all non-cross-resistant drugs efficacious in this disease, to be administered shortly before 131-I-MIBG. This radio-active drug is known to be effective in NB-resistant patients. For our heavily pretreated patients, the therapeutic results seem to be very encouraging in terms of degree and rapidity of response after only a single course of multiple drug chemotherapy in combination with 131-I-MIBG. In similar patients treated with intensive chemotherapy or 131-I-MIBG alone, therapeutic results are less impressive and usually obtained after a number of treatment courses administered over several months (Philip et al, 1987; Pinkenton et al, 1990; Frappaz et al, 1992; Dekraker et al, 1995). Our results may be partly due to the potential synergism between cisplatin, included in the CO-TH, which is apparently still present in tumour tissue for long periods after its administration, and radiation from 131-I-MIBG administration.

**Table 4** Tumor (T)/Background (B) 131-I-MIBG uptake ratio before and after chemotherapy

| | T/B at 24 h | | T/B at 48 h | |
|---|---|---|---|---|
| | **Pre** | **Post** | **Pre** | **Post** |
| Case n. 10[R] | 1,35 | 1,5 | 1,5 | 1,9 |
| Case n. 1 | 2,4 | 2,7 | 3,6 | 3,5 |
| Case n. 2 | 2,1 | 2,7 | 2,15 | 2,9 |
| Case n. 4 | 4,8 | 4,8 | 7,0 | 7,1 |
| Case n. 5 | 1,95 | 1,98 | – | – |
| Case n. 16[R] | 1,66 | 1,66 | – | – |

Due to the inaccuracies of dosimetric calculations in advanced NB (Beierwaltes, 1987), neither tumour nor whole-body radiation dosimetry was attempted. Instead, we administered a substantial single dose of 131-I-MIBG – approximately 200 mCi – using a similar dose in all the patients studied. This 131-I-MIBG dose was chosen because it is considered to be effective in NB and apparently safe when administered alone (Dekraker et al, 1995).

Tumour dose absorption may theoretically have been reduced in our cases as a consequence of chemotherapy, possibly because of a

*© 2001 Cancer Research Campaign* 

less active 131-I-MIBG uptake secondary to a decreased number of metabolically competent tumour cells. However, observations both ‘in vitro’ and ‘in vivo’ in xenograft models (Meco et al, 1997; Riccardi, unpublished observations) and in 6 of our patients, showed no evident alteration in MIBG uptake (Table 4). In fact, it is worth noting that NB cells, pre-incubated with cisplatin, concentrate MIBG more efficiently than untreated NB cells (Armour et al, 1997).

Our primary concern was haematological and non-haematological toxicity as a result of this new therapeutic regimen. In our previous work with a different CO-TH regimen a prolonged haematological toxicity was observed (Mastrangelo et al, 1995; Mastrangelo et al, 1997). In the present investigation the rationale for the drugs plus 131-I-MIBG sequence rests on experimental work in a murine model. Whereas, in mice, Cy administered after total body irradiation (TBI) is associated with severe haematological toxicity, it was found that pre-treatment with Cy significantly reduced TBI-induced mortality, perhaps by enhancement of BM recovery rather than by stem cell protection (Millar and Hudspith, 1976; Yan et al, 1991). Tumour tissue is not protected by Cy pretreatment. Subsequently, pretreatment with other drugs such as ARA-C and Vinblastine, in addition to Cy, appeared to protect mice from an otherwise lethal dose of radiation, suggesting that a common biological mechanism may be involved, such as rapid myeloid cell destruction (Millar et al, 1971). The optimum interval between administration of these drugs and radiation is 2–3 days, but we empirically chose a longer interval between chemotherapy and 131-I-MIBG administration, in consideration of different human haematopoiesis. CO-TH was well tolerated with this regimen. Our resistant, heavily pre-treated patients showed an acceptable haematological toxicity. A severe haematological toxicity was observed in 3 cases, possibly because melphalan, a drug which specifically causes stem cell damage, had been administered in a previous therapeutic regimen about 4–6 weeks before CO-TH. When a nadir for platelets and neutrophils might be expected from 131-I-MIBG, i.e. during the 4th and 5th weeks (Lashford et al, 1992), there was already a trend toward haematological recovery in the majority of patients studied. It is therefore suggested that the limited haematological toxicity observed was due to a ‘priming effect’ of the chemotherapy even though many patients did not have bone marrow involvement. It is interesting to note that there was no extra-haematological toxicity in any of the patients.

On the basis of these encouraging results and, above all, the acceptable haematological toxicity in resistant patients, we feel that CO-TH could be a promising therapeutic approach to use at the time of diagnosis.

Although they are preliminary, the results of CO-TH therapy in the 5 patients with advanced NB treated at diagnosis suggest that this regimen is very efficacious even after a single course of treatment.

In conclusion, while this is an ongoing study it already demonstrates that our innovative CO-TH therapy is feasible and most effective in obtaining a rapid response in heavily pre-treated resistant patients and in patients treated at diagnosis. In the latter, considering that only mild haematological toxicity occurred, a carefully monitored dose-escalation of CO-TH could be contemplated with the aim of rapidly eradicating the maximum number of tumour cells before resistance is acquired.

### ACKNOWLEDGEMENTS

This work was supported by grants from the Fondazione per l’Oncologia Pediatrica.

### REFERENCES

Armour A, Cunnigham SH, Gaze MN, Wheldon TE and Mairs RJ (1997) The effect of cisplatin pretreatment on the accumulation of MIBG by neuroblastoma cells in vitro. *Br J Cancer* **75**: 470–476

Beierwaltes WH (1987) Treatment of neuroblastoma with 131-I-MIBG: dosimetric problems and perspective. *Med Ped Oncol* **15**: 188–191

Brodeur GM, Pritchard J, Berthold F, et al (1993) Revision of the International Criteria for Neuroblastoma diagnosis, staging and response to treatment. *J Clin Onc* **11**: 1466–1477

DeKraker J, Hoefnagel CA, Caron H, et al (1995) First line targeted radiotherapy, a new concept in the treatment of advanced stage neuroblastoma. *Eur J Cancer* **31A**: 600–602

Frappaz D, Michon J, Hartmann O, et al (1992) Etoposide and carboplatin in neuroblastoma: a French Society of Pediatric Oncology phase II study. *J Clin Oncol* **10**: 1592–1601

Geatti O, Shapiro B, Sisson JC, et al (1985) Iodine-131 metaiodobenzylguanidine for the location of neuroblastoma: Preliminary experience in ten cases. *J Nucl Med* **26**: 736–742

Iavarone A, Lasorella A, Servidei T, et al (1993) Uptake and storage of *m*-iodobenzylguanidine by neuroblastoma cells results from indipendent uptake and storage mechanism. *Cancer Res* **51**: 4342–4346

Lashford LS, Lewis iJ, Fielding SL, et al (1992) Phase I/II study of iodine 131-I-Metaiodobenzylguanidine in chemoresistant neuroblastoma: a United Kingdom Children’s Cancer Study Group investigation. *J Clin Oncol* **10**: 1889–1896

Mastrangelo R (1987) Editorial: The treatment of neuroblastoma with $^{131}$I-MIBG. *Med Ped Oncol* **15**: 157–158

Mastrangelo R and Voute PA (1991) Session on the treatment of neuroblastoma with radioiodinated metaiodobenzylguanidine. Chairmen’s Report. *J Nucl Biol Med* **35**: 260–262

Mastrangelo R, Troncone L, Lasorella A, Riccardi R, Montemaggi P and Rufini V (1989) $^{131}$I-MIBG in the treatment of neuroblastoma at diagnosis. *J Ped Hematol Oncol* **11**: 28–31

Mastrangelo R, Lasorella A, Iavarone A, et al (1993) Critical Observation on neuroblastoma treatment with 131-I-metadiodobenzylguanidine at diagnosis. *Med Ped Oncol* **21**: 411–415

Mastrangelo R, Tornesello A, Riccardi R, et al (1995) A new approach in the treatment of stage IV neuroblastoma using a combination of [131I]metaiodobenzylguanidine (MIBG) and cisplatin. *Eur J Cancer* **31A(4)**: 606–611

Mastrangelo R, Tornesello A, Lasorella A, et al (1997) Optimal use of 131-I-metaiodobenzylguanidine and cisplatin combination in advanced neuroblastoma. *J Neurooncol* **31** (1–2): 153–158

Matthay KK, Villablanca JG, Seeger RC , Stram DO, et al (1996) Treatment of high risk neuroblastoma with intensive chemotherapy, radiotherapy, autologous bone marrow transplantation, and 13-cis-retinoid acid *N Engl J Med* **341**: 1165–1173

Matthay KK, DeSantes K, Hasegawa B, Huberty J, Hattner RS, Ablin A, Reynolds CP, Seeger RC, Weinberg VK and Price D (1998) Phase I dose escalation of $^{131}$I-Metaiodobenzylguanidine with autologous bone marrow support in refractory neuroblastoma. *J Clin Onc* **16**: 229–236

Meco D, Lasorella A, Riccardi A, Rumi C, Riccardi R, Mastrangelo R, et al (1997) Chemotherapy and MIBG uptake in neuroblastoma cell lines. SIOP XXIX Meeting, Istanbul, *Med Ped Oncol* **29**: 374

Millar JL, Hudspith BN, et al (1976) Sparing effect of cyclophosphamide (NSC-26271) pretreatment on animals lethally treated with $\gamma$-irradiation. *Cancer Treat Rep* **60**: 409–414

Millar JL, Blacker NM, Hudspith BN, et al (1971) Enhanced post-irradiation recovery of the hematopoietic system in animals pretreated with a variety of cytotoxic agents. *Cell Tissue Kinet* **11**: 543–553

Philip T, Ghalie R, Pinkerton R, et al (1987) A phase II study of high-dose cisplatin and VP-16 in neuroblastoma: a report from the Société Française d’Oncologie Pédiatrique. *J Clin Oncol* **5**: 941–950

Pinkerton R, Zucher JM, Hartmann O, et al (1990) Short duration, high dose, alternating chemotherapy in metastatic neuroblastoma (ENSG 3C induction regimen). *Br J Cancer* **62**: 319–323

Riccardi R. Unpublished observations

Simone JV (1984) The treatment of neuroblastoma. *J Clin Oncol* **2(7)**: 717–718

Wieland DM, Wu J-L, Brown LE, et al (1980) Radiolabeled adrenergic neuron-labeling agents: Adrenomedullary imaging with $^{131}$Iliodobenzylguanidine. *J Nucl Med* **21**: 349–353

World Health Organization (1979) WHO Handbook for Reporting Results of Cancer Treatment. WHO offset publication no. 48. Geneva, Switzerland, World Health Organization

Yan R, Peters LJ, Travis EL, et al (1991) Cyclophosphamide 24 hours before or after total body irradiation: effect on lung and bone marrow. *Radioth Oncol* **21**: 149–156

 *© 2001 Cancer Research Campaign*
